# The establishment of a duplex real-time PCR assay for rapid and simultaneous detection of *bla*_NDM_ and *bla*_KPC_ genes in bacteria

**DOI:** 10.1186/1476-0711-12-30

**Published:** 2013-10-22

**Authors:** Fen Zheng, Jingjing Sun, Cancan Cheng, Yongyu Rui

**Affiliations:** 1 Laboratory Medicine Center, Nanfang Hospital, Southern Medical University, Guangzhou 510515, China

**Keywords:** Duplex, Real-time PCR, Infection control, Carbapenemase

## Abstract

The latest threat of multidrug-resistant Gram-negative bacteria corresponds to the emergence of carbapenemase New Delhi metallo-β-lactamase (NDM) and *Klebsiella pneumoniae* carbapenemase (KPC) producers. Rapid molecular detection is essential to limit their spread. In this study, a duplex real-time polymerase chain reaction (PCR) that was specific for the detection of *bla*_NDM_ and *bl*a_KPC_ with the same limit of detection of ten plasmid copies was developed. The assay was linear over eight log dilutions for *bla*_NDM_ (R^2^ = 0.971; slope, -3.273) and *bla*_KPC_ (R^2^ = 0.992; slope, -2.997) with efficiencies of 102% and 115%, respectively. The assay was validated with 157 clinical isolates and showed 100% concordance with conventional PCR. The excellent performance of the duplex PCR assay makes it a powerful tool for surveillance of the carbapenemases NDM and KPC.

## Introduction

Carbapenems such as imipenem and meropenem are considered the drugs of choice for treating severe infections caused by *Enterobacteriaceae*-producing extended-spectrumβ-lactamases (ESBLs). Increasing resistance to carbapenems is now frequently observed in many hospital-acquired and community-acquired *Enterobacteriaceae* rods [1,2]. The association of carbapenem-resistant *Enterobacteriaceae* (CRE) with therapeutic failure and the resulting high mortality rates is of serious concern. Although several mechanisms of carbapenem resistance have been reported, the production of carbapenemases is the most common mechanism [2]. A large variety of clinically relevant carbapenemases, including Guiana extended-spectrum β-lactamases (GES), *Klebsiella pneumoniae* carbapenemase (KPC), Verona integron-encoded metallo-β-lactamase (VIM), imipenem-hydrolyzing β-lactamases (IMP), Sao Paulo metallo-β-lactamase (SPM), German imipenemase (GIM), Seoul imipenemase (SIM), and oxacillinase-48 (OXA-48) type, and rapidly emerging New Delhi metallo-β-lactamaseo-1 (NDM-1) have been reported [1,3]. In particular, since KPC producers were first reported in the United States in 1996 and NDM producers were first reported in India in 2008, both have rapidly emerged worldwide as important causes of extreme drug resistance [3-5].

The increasingly common class A enzyme KPCs and the latest metallo-β-lactamase NDMs are able to hydrolyze nearly all β-lactams; moreover, almost all of their producers are broadly resistant to other drug classes in addition to β-lactams due to a diversity of other resistance mechanisms (e.g. to aminoglycosides and fluoroquinolones), leaving physicians with limited antibiotic choices for treating infected patients [1,6,7]. KPC enzymes have been reported in numerous *Enterobacteriaceae* genera and even in non-fermenting bacteria, although KPCs are mostly identified from *K*. *pneumoniae*. In addition, KPC-producing isolates have disseminated worldwide, including several countries across Asia, America and Europe. The *bla*_KPC_ genes are almost present on transferable plasmids and are flanked by transposable elements [8-10], which allows for potential dissemination among different species. Similarly, the *bla*_NDM_ genes are primarily located on plasmids belonging to several incompatibility groups, and the diversity of genetic features associated with the *bla*_NDM_ genes may explain its current high worldwide spread rate [11,12]. NDM enzymes have been recognized among different *Enterobacteriaceae* species as well as in non-fermenters and *Vibrionaceae* and have been reported worldwide in Asia, Europe, North America, Australia and the Middle East since 2008. To date, thirteen *bla*_KPC_ gene variants (classified in sequential numeric order from *bla*_KPC-1/2_ to *bla*_KPC-15_) have been described and six minor NDM variants (bla_NDM-2_ to bla_NDM-8_) have been identified (http://www.lahey.org/studies/).

The carbapenemases KPC and NDM have already become a worldwide public health issue due to their widespread distribution, broad range of activity against β-lactams and aggressive hazards to human beings. As a result, the sensitive and rapid detection of the *bla*_NDM_ and *bla*_KPC_ genes is essential for implementation of the infection control procedures that are required to limit their spread and to help clinicians guide individual patient management. In this study, we developed a duplex TaqMan probe-based real-time polymerase chain reaction (PCR) assay for the prompt and simultaneous detection of the *bla*_NDM_ and *bla*_KPC_ genes in bacteria in a single tube.

## Materials and methods

### Primer and probe design

The details of the reference genes used in this assay are provided at http://www.lahey.org/studies/. The reference gene sequences for the NDM and KPC enzyme families were assembled from GenBank (http://www.ncbi.nlm.nih.gov/GenBank) as NDM accession numbers FN396876, JF703135, JQ734687, JQ348841, JN104597, JN967644, JX262694 and AB744718 representing alleles 1–8 and as KPC accession numbers AY034847, AF395881, AY700571, EU400222, EU555534, EU729727, FJ234412, FJ624872, GQ140348, HM066995, HQ641421, HQ342890, JX524191 and KC433553 representing alleles 2–15. Based on the comprehensive analyses and alignments of both gene families, primers and probes for the duplex real-time PCR assay were specifically designed to amplify all alleles of each gene family described above using Beacon Designer software (Premier Biosoft, Palo Alto, CA, USA) (Table 1). All of the primers and probes were synthesized by TaKaRa Bio, Inc. (Dalian, China).

**Table 1:** Primers and probes used in duplex real-time PCR

**Target**	**Primer/probe name**	**Sequence(5’-3’)**	**Position**	**Amplicon size (bp)**
*bla*_NDM_	NDM-F	TTGGCGATCTGGTTTTCC	137–154	195
	NDM-R	GGTTGATCTCCTGCTTGA	331–314	
	NDM-probe	JOE-TGGCAGCACACTTCCTATCTCG- ECLIPSE	175–196	
*bla*_KPC_	KPC-F	CGCAACTGTAAGTTACCG	162–179	187
	KPC-R	CATGCCTGTTGTCAGATA	348–331	
	KPC-probe	FAM –CCACTGTGCAGCTCATTCAAGG - ECLIPSE	196-217	

### Recombinant plasmids pNDM-1 and pKPC-2

The recombinant plasmids pNDM-1 and pKPC-2, composed of a pMD18-T backbone carrying a 4-kb insert expressing NDM-1 and KPC-2 carbapenemase, respectively, were used to optimize the quantitative PCR (qPCR).

### Bacterial strains and antibiotic susceptibility testing

A total of 157 *Enterobacteriaceae* isolates were collected at Nanfang Hospital from June 2010 to September 2012, including 17 resistant to imipenem or meropenem isolates and 140 randomly collected isolates with susceptible to imipenem and meropenem. All the isolates had previously been tested for *bla*_KPC_, *bla*_IMP_, *bla*_VIM_, *bla*_NDM_ and *bla*_OXA-48_ using conventional PCR and sequencing [13-15]. Of them, 17 carbapenem–resistant isolates harbored the carbapenemase genes *bla*_KPC_, *bla*_IMP_ or *bla*_NDM_, including nine *bla*_KPC-2_-positive *K*. *pneumoniae*, one *K*. *pneumoniae* carrying *bla*_KPC-2_ and *bla*_IMP-4_, three *bla*_NDM-1_-positive *K*. *pneumoniae*, one *Klebsiella oxytoca* and one each *bla*_NDM-1_-positive *Enterobacter hormaechei*, *Enterobacter cloacae* and *Enterobacter aerogenes*. The other 140 Enterobacteriaceae isolates were negative for the five tested carbapenemase genes. In addition, ten NDM/KPC non-producers, including *Escherichia coli* ATCC 25922, *K*. *pneumoniae* ATCC 700603, *Staphylococcus aureus* ATCC 25923, *Pseudomonas aeruginosa* ATCC 27853 and six known clinical strains without any carbapenemase gene(two *Enterococcus* spp., two *Streptococcus* spp. and two *Staphylococcus* spp.), were used to evaluate the specificity of the duplex qPCR assay.

All bacterial identification and susceptibility testing was performed using a BD Phoenix 100 Automated Microbiology System (Benton, Dickinson and Co., Franklin Lakes, NJ, USA). The results were interpreted according to the Clinical and Laboratory Standards Institute guidelines. The isolates were stored at -80°C in nutrient broth containing 30% (v/v) glycerol.

### DNA extraction

Fresh, well-isolated colonies were used for total bacterial DNA extraction using a TaKaRa MiniBEST DNA Fragment Purification Kit Ver.3.0 (TaKaRa Bio, Inc.) according to the manufacturer’s protocol. Extracted DNA was eluted from the columns in 100 μL of elution buffer and stored at -20°C.

### Duplex real-time PCR reaction

The TaqMan probe-based real-time PCR assay was performed on an ABI Prism 7500 Fast apparatus (Applied Biosystems, Foster City, CA, USA) using the Premix *Ex Taq*™ (Probe qPCR) kit (TaKaRa Bio, Inc.) as recommended by the manufacturer. The 20-μL qPCR mixture contained 2 μL of DNA extract solution, 10 μL of Premix Ex Taq Probe qPCR (2×), 0.4 μL of ROX Reference Dye II (50×), 0.2 μM of each primer and 0.1 μM of each TaqMan probe, and sterile water to a final volume of 20 μL. The optimal cycling conditions were 20 s at 95°C and 40 cycles each of 3 s at 95°C and 30 s at 60°C. Each run contained a positive control (mixture of recombinant plasmids pNDM-1 and pKPC-2) for *bla*_NDM_ and *bla*_KPC_ amplification and at least one water blank as a negative control.

### Standard curve and sensitivity test on recombinant plasmids

To determine the efficiency of the duplex real-time PCR assay, Ct values obtained from a series of template DNA dilutions were graphed on the y axis versus the log of the dilution on the x axis. The slope of this line was used to determine efficiency (E) according to this equation: E = 10^(-1/slope)^. Assay sensitivity and reproducibility were estimated by serial ten-fold dilution experiments using the mixture of recombinant plasmids pNDM-1 and pKPC-2.

## Results

The assay was initially validated using the recombinant plasmids pNDM-1 and pKPC-2 carrying the *bla*_NDM-1_ and *bla*_KPC-2_ genes. Clearly defined amplification curves were observed for the two positive recombinant plasmids in their predicted fluorescence channels.

Primer and probe specificities were evaluated at the National Center for Biotechnology Information database (http://www.ncbi.nlm.nih.gov). No matches to the primer or probe sequences of *bla*_NDM_ and *bla*_KPC_ were found other than those for corresponding *bla*_NDM_ and *bla*_KPC_ genes. The analytical specificity was also applied on clinically representative bacteria with no known antibiotic resistance gene, including *E*. *coli* ATCC 25922, *K*. *pneumoniae* ATCC 700603, *S*. *aureus* ATCC 25923, *P*. *aeruginosa* ATCC 27853, *Enterococcus* spp. (two isolates), *Staphylococcus* spp. (two isolates) and *Streptococcus* spp. (two isolates). The recombinant plasmids pNDM-1 and pKPC-2 were used as positive controls. No amplification of *bla*_NDM_ or *bla*_KPC_ was observed with DNA extracted from any of the ten organisms on our specificity test panel. Thus, the specificity of our duplex PCR is considered satisfactory.

**Figure 1:**
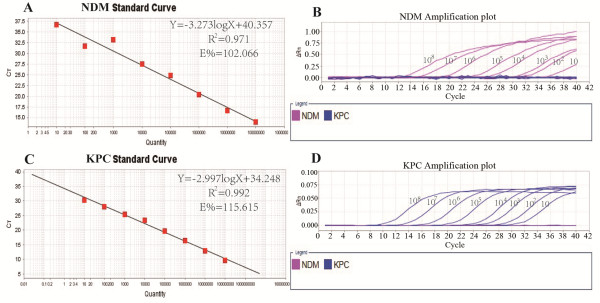
**Duplex real-time PCR 10-fold dilution series.** E: Efficiency; R^2^: coefficient of determination. Standard curves (Ct plotted against the log of the starting quantity of template for each dilution) and amplification curves of the dilution series for **(A, B)***bla*_NDM_ and **(C, D)***bla*_KPC_.

Assay linearity and limit of detection were determined by performing serial ten-fold dilutions of a mixture of the recombinant plasmids pNDM-1 and pKPC-2 from 10 to 10^8^ copies/μL (Figure 1). The assay correlated well with *bla*_NDM_ (R^2^ = 0.971) and *bla*_KPC_ (R^2^ = 0.992) over the entire copy range with an efficiency of 102% and 115%, respectively. The limit of detection for both targets was 10 copies per 20-μL reaction volume.

We have also shown that the assay was highly stable and precise as evidenced by the performance of the recombinant plasmid pNDM-1 (10^5^ copies of *bla*_NDM-1_-carrying plasmid) and pKPC-2 (10^5^ copies of *bla*_KPC-2_-carrying plasmid), which were stable over five consecutive runs for both *bla*_NDM_ (mean Ct, 22.8; standard deviation, 0.4; coefficient of variation, 1.8%) and *bla*_KPC_ (mean Ct, 19.3; standard deviation, 0.1; coefficient of variation, 0.5%).

Finally, when tested against the 157 previously characterized carbapenem-resistant Enterobacteriaceae isolates, including seven with NDM-1 metallo-carbapenemases, ten with KPC-2 serine carbapenemases and 140 strains without *bla*_NDM_ and *bla*_KPC_ carbapenemases, the duplex PCR assay showed 100% concordance with conventional PCR previously identified. In our opinion, the 100% sensitivity and specificity of the duplex PCR validated against clinical isolates of carbapenem-resistant Gram-negative bacilli with well-defined carbapenemase genes make it a useful tool for the screening and surveillance of isolates carrying the carbapenemase genes *bla*_NDM_ and *bla*_KPC_.

## Discussion

The co-occurrence of *bla*_NDM-1_ and *bla*_KPC-2_ in a clinical isolate of *K*. *pneumoniae* from India was recently reported [7], suggesting that the growing emergence of these powerful resistance mechanisms should be given great attention. Considering the difficulty in preventing their emergence due to the potential for rapid horizontal and vertical transmission determined by genetics and treating patients infected with *bla*_NDM_ and *bla*_KPC_ harboring bacterial pathogens, active surveillance and early detection are mandatory for preventing their further spread.

Molecular assays have been reported to identify carbapenemase genes in Gram-negative bacteria. Several real-time PCR assays targeting NDM-1 or KPC have been described [16-20], but each targets only one kind of carbapenemase gene. Assays that target more than one class of carbapenemase (A, B, D) have been developed [15,21], but each of those uses conventional PCR requiring post-amplification analysis. Real-time PCR requiring less time and labor for the simultaneous detection of multiple carbapenemase genes including NDM and KPC is rare. Monteiro et al. described a multiplex real-time PCR for the rapid detection of KPC, GES, NDM, IMP, VIM and OXA-48 carbapenemase genes using melt curve analysis [22], however, no details of the assay design for the detection of all *bla*_NDM_ types other than *bla*_NDM-1_ were provided. The presently described duplex PCR assay is capable of simultaneously detecting all of the minor *bla*_NDM-1_ variants since the nucleotide changes between the variants are located outside the primer- and probe-binding sequences. Likewise, in the case of *bla*_KPC_, the assay described here is expected to amplify all known *bla*_KPC_ types. Although our duplex PCR assay was validated with Gram-negative bacteria, it could amplify the expected product from all *bla*_KPC_- and *bla*_NDM_-carrying isolates, including Gram-positive bacteria. Recently, Cunningham SA et al. described a FRET hybridization probe-based real-time PCR assay that targets *bla*_KPC_ and *bla*_NDM_ in a single assay [23]. Although the assay performed equivalently to our assay, the FRET hybridization probe is more expensive than ours TaqMan probe. Additionally, the FRET hybridization probe-based real-time PCR assay needs LightCycler instrument(Roche Applied Science, Indianapolis, IN) to match it, which limited its wide application; the classic TaqMan probe in our assay is suitable for any real-time PCR detection system, which has wider applicability.

In conclusion, we successfully established a novel duplex real-time PCR assay for the prompt and simultaneous screening of the *bla*_NDM_ and *bla*_KPC_ genes in a single reaction that had good sensitivity and specificity and excellent agreement with conventional PCR and sequencing. The excellent performance of duplex PCR makes it an important tool for guiding the appropriate choice of antimicrobial therapy and helping limit spread of the resistance genes through aggressive infection control measures. Its use would be especially suitable for national epidemiological purposes in an outbreak situation thanks to its comprehensive ability to detect all known *bla*_NDM_ and *bla*_KPC_ variants.

### Nucleotide sequence accession number

The GenBank accession numbers are JN711113, JN711114, KC404861, KC539432, KC539429, KC539430 and KC539431, for the *bla*_NDM-1_ gene from strains 920856, 903966, 917144, 23142, 24192, 27378 and 208741, respectively, and JF431928, JF894295-JF894300, JQ040039, and JQ040040 for the *bla*_KPC-2_ gene from nine KPC-2-producing *K*. *pneumonia*, respectively. The *bla*_KPC-2_ gene of One *K*. *pneumonia* carrying both *bla*_KPC-2_ and *bla*_IMP-4_ genes did not been lodged in GenBank.

## Competing interests

The authors declare that they have no competing interests.

## Authors’ contributions

FZ and JS carried out the design of the primers and probes, the evaluation experiments, data organization and analysis and contributed to writing and to the interpretation of the results. CC carried out duplex real-time PCR assay and the sequence alignment. YR contributed to the design of the study and assisted in the drafting of the manuscript. All authors have read and approved the manuscript.
